# Post-Hypospadias Repair Penile Score in Follow-Up Patients of Urethroplasty

**DOI:** 10.7759/cureus.23816

**Published:** 2022-04-04

**Authors:** Anum Manzoor, Nabila Talat, Hafiz Muhammad Adnan, Muhammad Zia, Muhammad Ahsen Aziz, Ezza Ahmed

**Affiliations:** 1 Pediatric Surgery, The Children’s Hospital and University of Child Health Sciences, Lahore, PAK

**Keywords:** urethral meatus, uretheroplasty, surgical site infection, edema, hypospadias

## Abstract

Background: Hypospadias is described as the abnormal location of the urethral meatus upon the ventral surface of the penis with variable association with the abnormal development of the urethral spongiosum, ventral prepuce, and penile chordee. Numerous studies have utilized techniques like "Penile Perception Score (PPPS)," "Hypospadias Objective Scoring System (HOSE)," and "Hypospadias Objective Penile Evaluation Score (HOPE)" to evaluate the outcome after hypospadias repair, but there is a lack of evidence analyzing the utility of "Post-Hypospadias Repair Penile Score (PHRPS)." This study was carried out to assess PHRPS in children undergoing hypospadias repair.

Methodology: A prospective cohort study was conducted at the department of pediatric surgery, uni-II, The Children’s Hospital and University of Child Health Sciences, Lahore, Pakistan from November 2020 to December 2021. All male children aged up to 12 years and planning to undergo hypospadias repair during the study period were included. The PHRPS system was used to assess the outcomes of hypospadias repair. Qualitative data were represented as frequency and percentage, while mean and standard deviation (SD) were calculated for quantitative data.

Results: During this period, a total of 37 children were treated for hypospadias. The mean age of the patients at the time of repair was 8.2±3.6 years, ranging between 1.5 years and 12 years. Out of 37 patients, 14 (37.8%) had penoscrotal hypospadias, while 12 (32.4%) had distal penile hypospadias. In terms of acute post-surgery complications, edema was reported in 9 (24.3%), bleeding 1 (2.7%), and surgical site infection 1 (2.7), while all of these were successfully managed conservatively. Seven patients were lost to follow-up, so they were excluded from the final analysis. On the basis of PHRPS scoring, 17 (56.7%) patients had excellent outcomes, 2 (6.7%) had good outcomes, 8 (26.7%) had acceptable outcomes, and 3 (10.0%) had poor outcomes.

Conclusion: The PHRPS is a new but simple objective tool, facilitating surgical audit and balanced evaluation of the outcomes of traditional and innovative procedures. The outcome of hypospadias repair was generally found to be good.

## Introduction

Hypospadias is described as the abnormal location of the urethral meatus upon the ventral surface of the penis with variable association with the abnormal development of the urethral spongiosum, ventral prepuce, and penile chordee. As per meatal location, hypospadias is categorized as anterior (glanular and subcoronal) contributing to 50% of cases, mid-penile (distal penile, midshaft, and proximal penile) contributing to 30%, or posterior (penoscrotal, scrotal, and perineal) accounting for 20% of cases. It is estimated that around 1/4th of the hypospadias cases are linked with chordee [1]. Hypospadias is calculated to affect between 0.3% and 0.4% of the male population globally [2]. In recent decades, increasing trends have been observed in the number of hypospadias surgeries performed in different parts of the world, but despite improvements in surgical techniques, nearly half of the cases undergoing hypospadias surgeries report some kind of post-surgical complications [3,4].

Most of the research performed analyzing various aspects of hypospadias consists of observational or retrospective studies, while there is no consensus about the most suitable technique or post-surgical assessment tool in cases of hypospadias repair. Each post-surgical assessment tool has its own advantages and disadvantages. The most commonly used assessment tools [5,6] are "Penile Perception Score (PPPS)," "Hypospadias Objective Scoring System (HOSE)," and "Hypospadias Objective Penile Evaluation Score (HOPE)" to evaluate the outcome after hypospadias repair, but there is a lack of evidence analyzing the utility of "Post-Hypospadias Repair Penile Score (PHRPS)."

A study carried out by El-Sawaf recently introduced PHRPS as a new simple objective tool that facilitated auditing of surgical outcomes. The statistical correlation between PHRPS and experts' opinions implies its superiority for accurate post-operative evaluation of hypospadias surgery outcomes [7]. Numerous studies have utilized techniques like PPPS, HOSE, and HOPE to evaluate the outcome after hypospadias repair, but there is a lack of studies that utilized PHRPS. Hence, this study was carried out to assess PHRPS in children undergoing hypospadias repair.

## Materials and methods

Study design, place, and duration

A prospective cohort study was conducted at the Department of Pediatric Surgery, unit-II, The Children’s Hospital and University of Child Health Sciences, Lahore, Pakistan from November 2020 to December 2021.

Inclusion and exclusion criteria

All male children aged up to 12 years and planning to undergo hypospadias repair during the study period were included. Patients with comorbidities like coagulation disorders, diabetes mellitus, or disorders of sex development and those patients whose parents/guardians refused to be part of the study were excluded.

Data collection

Approval from the Institutional Review Board (letter-number CHICH/19-32) was acquired. Written consent was obtained from the parents/guardians. A single-staged procedure, Snodgrass repair, was done in patients with distal penile and mid-penile hypospadias. A staged procedure was done in patients with proximal penile hypospadias (out of 14 patients, 1 underwent the Byars procedure and 13 underwent the BRACKA procedure). The catheter was kept in place for 7 to 10 days. The PHRP system has eight parameters, including (1) post-operative meatal location; (2) post-operative meatal shape; (3) urine stream; (4) curvature during erection, (5) fistula, (6) penile skin, (7) shape of the glans, and (8) rotation. Scores between 18 and 23 are labeled as excellent, 13-17 are acceptable, and 7-12 are poor, while scores below or equal to 6 are termed as crippled. A special proforma was prepared to record all the study data.

Data analysis

For data analysis, "Statistical Package for the Social Sciences (SPSS)" version 26.0 (IBM Corp., Armonk, NY) was used. Qualitative data were represented as frequency and percentage, while mean and standard deviation (SD) were calculated for quantitative data.

## Results

During the study period, a total of 37 children were treated for hypospadias. The mean age of the patients at the time of repair was 8.2±3.6 years, ranging between 1.5 years and 12 years. Two (5.4%) children had a family history of hypospadias. Out of 37 patients, 14 (37.8%) had penoscrotal hypospadias, while 12 (32.4%) had distal penile hypospadias.

**Figure 1:**
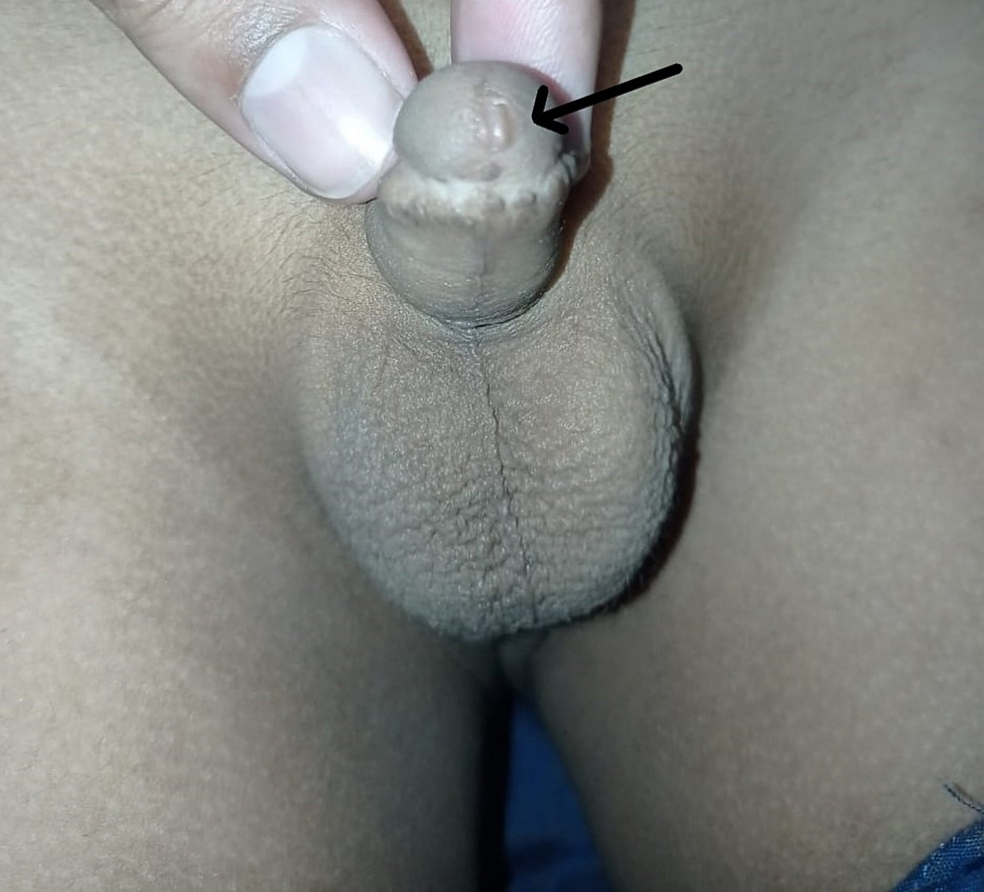
Excellent outcome as per Post-Hypospadias Repair Penile Score

In terms of acute post-surgery complications, edema was reported in 9 (24.3%), bleeding 1 (2.7%), and surgical site infection 1 (2.7), while all these were successfully managed conservatively. Seven patients were lost to follow-up, so they were excluded from the final analysis. On the basis of PHRPS scoring in the remaining 30 patients included in the final analysis, 17 (56.7%) had excellent outcomes, 2 (6.7%) had good outcomes, 8 (26.7%) had acceptable outcomes, and 3 (10.0%) had poor outcomes. Table 1 shows details of PHRPS with respect to outcomes. Figure 1 shows a picture of an excellent outcome for the patient as per PHRPS.

**Table 1:** Details of Post-Hypospadias Repair Penile Score with respect to outcomes (n=30)

Variable	Score	Results
Post-operative meatal location		
- Distal glanular	4	63.3% (19 patients)
- Proximal glanular	3	10.0% (3 patients)
- Coronal	2	6.7% (2 patients)
- Penile shaft	0	20.0% (6 patients)
Post-operative meatal shape		
- Vertical slit	2	63.3% (19 patients)
- Circular	1	33.3% (10 patients)
- Distorted	0	3.3% (1 patients)
Urine stream		
- Single stream	2	80.0% (24 patients)
- Spray	1	20.0% (06 patients)
- Multiple streams	0	-
Curvature during erection		
- Straight	3	40.0% (12 patients)
- Angulation <10º	2	36.7% (11 patients)
- Angulation 10–45º	1	23.3% (7 patients)
- Angulation >45º	0	-
Fistula		
- None	4	73.3% (22 patients)
- Single – sub-coronal or more distal	3	10.0% (3 patients)
- Single – proximal or mega fistula	1	13.3% (4 patients)
- Multiple or complex	0	3.3% (1 patient)
Penile skin		
- No scars or linear scar	3	36.6% (11 patients)
- Slight scarring and pumps	1	53.3% (16 patients)
- Severe scarring or disfigurement	0	10% (03 patients)
Shape of the glans		
- Acorn shape	3	60% (18 patients)
- Slightly disfigured	2	30% (09 patients)
- Flat or open glans	1	10% (03 patients)
- Severe disfigurement	0	-
Rotation		
- 0–30º	2	66.6% (20 patients)
- 30–70º	1	33.3% (10 patients)
- >70º	0	-

## Discussion

Outcomes following hypospadias surgery are aimed at producing a normal-looking penis, with the meatus at the tip, in a single-stage operation [8-10]. Factors like hypospadias grade, techniques of repair, skill sets of the surgeons, etc., are known to affect the outcomes of hypospadias surgery outcomes [11,12]. Some studies have reported that the higher the age at the time of repair, the higher the complications [13,14].

We noted the mean age of the patients was 8.2±3.6 years, which is not aligned with the guidelines for the timing of hypospadias repair, indicating the ideal time of hypospadias repair to be between 6 and 18 months [15,16]. The reason for the relatively higher age at the time of hypospadias repair could be mainly due to a lack of knowledge in the general public about this condition and financial reasons. Social stigma could be another reason for the late presentation of hypospadias in our study. In this study, penoscrotal hypospadias (37.8%) was the most frequent type of hypospadias, while 32.4% had distal penile hypospadias. Another local study revealed "mid-penile hypospadias" to be the commonest type, followed by anterior hypospadias [17]. Some other researchers have revealed anterior hypospadias to be the most commonly observed type [18,19]. Data from China found proximal hypospadias to comprise 65.7% of cases [20]. All these studies show that variation exists regarding the most frequent types of hypospadias around the globe.

In the present study, on the basis of PHRPS scoring, 56.7% had excellent outcomes, 6.7% had good outcomes, 26.7% had acceptable outcomes, and 10.0% had poor outcomes. So, overall, 90% of the patients reported acceptable to excellent outcomes. According to a study done by El-Sawaf analyzing 40 post-hypospadias repair children using PHRPS, the scores of each patient were expressed as a percentage of the maximal score possible. The study revealed that the PHRPS of patients ranged from 14 to 21 [21]. There was no statistical difference between the experts. The study concluded that PHRPS was an objective, simple, reproducible, and validated tool measuring all relevant and surgically correctable aspects of hypospadias [7].

Limitations of the study

Our study had some limitations as well. As this was a single-center study with a relatively small sample size, our findings cannot be generalized. We aimed to evaluate the PHRPS system in the present study, but we did not have any comparator group or randomization, so further studies comparing PHRPS with other contemporary systems comparing hypospadias repair need to be done. We noted relatively short follow-ups in this study, so studies assessing long-term follow-ups and utilization of PHRPS should be planned in the future.

## Conclusions

Post-Hypospadias Repair Penile score is a new but simple objective tool, facilitating surgical audit and balanced evaluation of the outcomes of traditional and innovative procedures. The outcome of hypospadias repair was generally found to be good. Postoperative functional and cosmetic long-term outcomes are usually comparable to those of a person not born with hypospadias.
